# Psychological well-being and physical activity before and after rotator cuff repair: a scoping review

**DOI:** 10.1016/j.xrrt.2026.100804

**Published:** 2026-06-19

**Authors:** Hadeel Y. Alghanim, Naif Z. Alrashdi, Megan Bell, Elroy J. Aguiar, Marcus A. Rothermich, Scott Snyder, Hon K. Yuen, Matthew P. Ithurburn

**Affiliations:** a Rehabilitation Science Program, University of Alabama at Birmingham, Birmingham, AL, USA; b Occupational Therapy Program, College of Applied Medical Sciences, King Saud bin Abdulaziz University for Health Sciences, Jeddah, Saudi Arabia; c King Abdullah International Medical Research Center, Jeddah, Saudi Arabia; d Department of Physical Therapy and Health Rehabilitation, College of Applied Medical Sciences, Majmaah University, AL-Majmaah, Saudi Arabia; e Health and Basic Sciences Research Center, Majmaah University, AL-Majmaah, Saudi Arabia; f UAB Libraries, University of Alabama at Birmingham, Birmingham, AL, USA; g Department of Kinesiology, The University of Alabama, Tuscaloosa, AL, USA; h Andrews Sports Medicine & Orthopaedic Center, Birmingham, AL, USA; i Department of Human Studies, School of Education, The University of Alabama at Birmingham, Birmingham, AL, USA; j Department of Occupational Therapy, University of Alabama at Birmingham, Birmingham, AL, USA; k American Sports Medicine Institute, Birmingham, AL, USA; l School of Health Professions, University of Alabama at Birmingham, Birmingham, AL, USA

**Keywords:** Orthopedic recovery, Shoulder surgery, Arthroscopy, Fear-avoidance beliefs, Pain, Patient-reported outcome measures (PROMs)

## Abstract

**Background:**

In patients with rotator cuff (RC) pathology, poor psychological well-being, such as depression and anxiety symptoms and kinesiophobia, has reported an association with worse shoulder symptoms, function, pain, and/or quality of life. Individuals with kinesiophobia often avoid certain activities, which may lead to muscle weakness, decreased range of motion, low physical activity (PA), and/or decreased function. This scoping review aimed to explore and summarize the current evidence regarding psychological factors and PA levels in patients with RC pathology before and after repair surgery.

**Methods:**

We searched PubMed, Embase, Scopus, SPORTDiscus, and PsycINFO databases for relevant studies. We included studies that enrolled patients with RC pathology undergoing repair surgery and reported psychological well-being and/or PA measures before and after surgery. We summarized the study characteristics and findings, including the study design, sample size, demographics, and measures of psychological well-being and/or PA before and after RC repair. Lastly, descriptive consolidation and qualitative synthesis were performed for the included studies.

**Results:**

Out of 11,026 potential studies, 13 studies met our criteria and were included. Of the studies included, 3 were retrospective and 10 were prospective studies, and all of them were published in 2015 and later. Among the included studies, depression symptoms (n = 10), anxiety symptoms (n = 7), kinesiophobia (n = 2), and negative thoughts (n = 2) were reported as psychological factors that negatively influence the symptoms and recovery patients with RC pathology before and after surgical repair. Importantly, none of the included studies examined PA measures in patients with RC pathology before and after RC repair.

**Conclusion:**

In patients with RC pathology, depression and anxiety symptoms have been commonly examined before and after RC repair. Worse depression and anxiety symptoms were reported to be associated with worse pain, reduced function, and lower quality of life. Consideration of PA in the screening and rehabilitation before and after RC repair was identified as a substantial gap in literature. Future research is encouraged to explore how the interplay between PA and psychological factors influences treatment outcomes for RC pathology, which may guide more holistic and effective rehabilitation strategies.

Rotator cuff (RC) pathology is a common degenerative shoulder condition.^22^ Importantly, symptomatic RC pathology often leads to significant shoulder pain, disrupted sleep, decreased shoulder functional performance (strength and range of motion [ROM]), and decreased quality of life (QoL).^4^^,^^9^^,^^63^ Nonoperative/conservative treatment for individuals with symptomatic RC pathology often focuses on relieving pain, improving function, and/or activity modification.^29^ However, for individuals with persistent symptoms or those unsatisfied with nonoperative management, RC repair surgery followed by rehabilitation is the gold standard intervention for restoring shoulder function and improving participation in activities of daily living.^29^ However, recovery from RC repair is multifactorial and may be influenced not only by physical impairments but also by psychological well-being and, potentially, engagement in physical activity (PA).^9^^,^^30^^,^^38^^,^^53^^,^^57^

Psychological well-being plays a role in the recovery of patients with different musculoskeletal injuries.^46^^,^^52^ Specifically, following RC repair, higher levels of depression and anxiety symptoms and negative pain beliefs associate with greater shoulder disability, worse pain, and decreased QoL.^18^^,^^40^ These psychological factors may potentially represent important targets for improving pain, adherence to rehabilitation, and/or functional recovery for patients undergoing RC repair.^26^^,^^51^

Besides psychological factors, PA engagement may also play a critical role in RC recovery. PA is “any bodily movement produced by skeletal muscles that requires energy expenditure,” defined by the World Health Organization.^77^ The World Health Organization recommends that adults (individuals aged 18–65 years) engage in at least 150–300 minutes of moderate PA (eg, walking briskly or raking the yard) or at least 75–150 minutes of vigorous PA (eg, jogging or carrying heavy groceries) per week.^78^ Regular engagement in PA facilitates injured tissue healing, enhances blood circulation, improves strength, and promotes overall health following major orthopedic injuries and surgeries.^7^^,^^42^^,^^74^ PA engagement also contributes to general health benefits, such as cardiovascular fitness and weight management.^6^ However, individuals who experience psychological distress may experience greater pain and disability, leading to decreased PA, strength, and functional performance.^32^ Beyond physical recovery, engaging in PA is also known to improve psychological well-being by reducing depression and anxiety symptoms and could contribute to higher QoL following orthopedic surgeries.^7^^,^^12^^,^^55^^,^^56^^,^^59^^,^^64^^,^^68^^,^^77^

Emerging evidence suggests that psychological well-being and PA may be inter-related.^7^^,^^74^ In individuals with RC pathology, those with movement-related fear, such as kinesiophobia and pain catastrophizing, may avoid certain activities or movements, which may in turn lead to muscle weakness, decreased ROM, decreased functional performance, and ultimately lower PA engagement.^45^ RC pathology may prevent patients from achieving the same level of functional performance as before pain/symptom onset, which may potentially lead to decreased PA^6^. On the other hand, participating in consistent PA could support optimal psychological well-being, improved mood, and psychological resilience.^77^ Taken together, a potential interplay between PA and psychological well-being may help shape recovery trajectories after RC repair surgery.^39^

Despite the growing recognition of the potential impact of PA engagement and psychological well-being on RC recovery trajectories and overall postsurgical health in patients undergoing orthopedic surgeries,^24^^,^^46^ there remains a substantial gap in the literature regarding their interplay following RC repair. Addressing this gap is critical as it may inform the development of comprehensive rehabilitation strategies related to psychological well-being as well as unique aerobic PA recommendations in patients undergoing RC repair. Therefore, this scoping review sought to comprehensively search for and summarize currently available evidence exploring PA levels and psychological well-being in patients with RC pathology before and after surgery, as well as the interplay between these 2 domains and their impact on postsurgical recovery.

## Methods

### Eligibility criteria for potential studies

In this scoping review, our eligibility criteria/search were structured according to the PECOS framework^49^: Table I demonstrates the PECOS framework, including Population, Exposure, Comparison, Outcomes, and Study design for this scoping review. We excluded conference proceedings abstracts, review studies, case studies (n-of-1 studies), non-English studies, or animal studies. Other studies that included any additional concomitant diseases/conditions/injuries along with RC pathology were also excluded.

**Table I:** PECOS framework.

PECOS component	Eligibility description
P: Population	Adults with rotator cuff pathology who have undergone surgical repair
E: Exposure/domains of interest	Psychological factors (eg, depression, anxiety, kinesiophobia, self-efficacy, pain catastrophizing) and/or physical activity levels assessed using self-reported or device-based measures
C: Comparison	Examined across presurgical and postsurgical time points
O: Outcomes	Post-operative outcomes including function and/or strength, pain, and quality of life
S: Study design	Observational and/or interventional study designs

### Information sources and search strategy

We used the framework proposed by Arksey and O'Malley to perform this scoping review.^3^ We also reported our study following the Preferred Reporting Items for Systematic Reviews and Meta-Analyses Extension for Scoping Reviews.^3^^,^^71^ Our search strategy was developed in collaboration with an academic research librarian (M.B.), who has expertise in searching medical literature databases. The librarian reviewed and refined our search terms before utilizing them in each database. Our search strategy was modified based on each database platform's command language, controlled vocabulary, and appropriate search fields using terms related to PA and psychological well-being in those with RC pathology undergoing RC repair (Table II).

**Table II:** Search strategy used for the current scoping review (adapted for other databases):

Database	Search Strategy
PubMed	("Rotator Cuff"[Mesh] OR "Rotator Cuff Injuries"[Mesh] OR "rotator cuff" OR "rotator cuff injuries" OR "rotator-cuff" OR "teres minor" OR "subscapularis" OR "infraspinatus" OR "supraspinatus") AND ("Psychological Well-Being"[Mesh] OR "Self-Concept"[Mesh] OR "Motivation"[Mesh] OR "Stress, Psychological"[Mesh] OR psychological∗ [tiab] OR self-efficac∗ [tiab] OR kinesiophobia∗ [tiab] OR confidence [tiab] OR anxiety∗ [tiab] OR stress∗ [tiab] OR emotional-wellbeing [tiab] OR emotional-well-being [tiab] OR Self-Perception∗ [tiab] OR Self-Esteem∗ [tiab] OR depress∗[tiab] OR "Depression"[Mesh] OR "Anxiety"[Mesh] OR anxiety∗[tiab] OR nervous∗ [tiab] OR "Fear"[Mesh] OR fear∗ [tiab] OR "Exercise"[Mesh] OR "Motor Activity"[Mesh] OR physical-activity∗ [tiab] OR exercise∗ [tiab]) AND ("Quality of Life"[Mesh:NoExp] OR "Quality of life" [tiab] OR "Sleep Initiation and Maintenance Disorders"[Mesh] OR sleep [tiab] OR insomnia∗ [tiab] OR "Pain"[Mesh] OR pain [tiab] OR "Muscle Strength"[Mesh] OR Strength [tiab] OR "Range of Motion, Articular"[Mesh] OR "Range of motion" [tiab] OR flexibility [tiab])
Embase	('rotator cuff':ti,ab,kw OR 'rotator cuff injuries':ti,ab,kw OR 'rotator-cuff':ti,ab,kw OR 'teres minor':ti,ab,kw OR 'subscapularis':ti,ab,kw OR 'infraspinatus':ti,ab,kw OR 'supraspinatus':ti,ab,kw OR 'rotator cuff'/exp OR 'rotator cuff injury'/exp) AND (psychological∗:ti,ab,kw OR 'self efficac∗':ti,ab,kw OR kinesiophobia∗:ti,ab,kw OR confidence:ti,ab,kw OR stress∗:ti,ab,kw OR 'emotional wellbeing':ti,ab,kw OR 'emotional well being':ti,ab,kw OR 'self perception∗':ti,ab,kw OR 'self esteem∗':ti,ab,kw OR depress∗:ti,ab,kw OR anxiety∗:ti,ab,kw OR nervous∗:ti,ab,kw OR fear∗:ti,ab,kw OR 'physical activit∗':ti,ab,kw OR exercis∗:ti,ab,kw OR 'psychological well-being'/exp OR 'self concept'/exp OR 'motivation'/exp OR 'physiological stress'/exp OR 'mental stress'/exp OR 'depression'/exp OR 'fear'/exp OR 'emotion'/exp OR 'anxiety'/exp OR 'physical activity'/exp OR 'physical activity, capacity and performance'/exp OR 'motor activity'/exp) AND ('quality of life':ti,ab,kw OR sleep:ti,ab,kw OR insomnia∗:ti,ab,kw OR pain:ti,ab,kw OR strength:ti,ab,kw OR 'range of motion':ti,ab,kw OR flexibility:ti,ab,kw OR 'quality of life'/exp OR 'sleep disorder'/exp OR 'sleep'/exp OR 'pain'/exp OR 'motor dysfunction'/exp OR 'muscle strength'/exp) AND ('article'/it OR 'article in press'/it OR 'review'/it)
Scopus	(TITLE-ABS-KEY (("rotator cuff" OR "rotator cuff injuries" OR "rotator-cuff" OR "teres minor" OR "subscapularis" OR "infraspinatus" OR "supraspinatus"))) AND (TITLE-ABS-KEY (("psychological well-being" OR "self concept" OR "motivation" OR "psychological stress" OR psychological∗ OR self-efficac∗ OR kinesiophobia∗ OR confidence OR anxiety∗ OR stress∗ OR "emotional wellbeing" OR "emotional well-being" OR "self perception" OR "self esteem" OR depress∗ OR "depression" OR anxiety∗ OR nervous∗ OR fear∗ OR exercise OR "physical activity"))) AND (TITLE-ABS-KEY (("quality of life" OR "sleep disorders" OR sleep OR insomnia∗ OR pain OR "muscle strength" OR strength OR "range of motion" OR flexibility))) AND (LIMIT-TO (DOCTYPE, "ar") OR LIMIT-TO (DOCTYPE, "re"))
PsycINFO	(rotator cuff OR "rotator cuff injuries" OR "rotator-cuff" OR "teres minor" OR "subscapularis" OR "infraspinatus" OR "supraspinatus") AND (MAINSUBJECT.EXACT.EXPLODE("Well Being") OR MAINSUBJECT.EXACT.EXPLODE("Self-Concept") OR MAINSUBJECT.EXACT.EXPLODE("Motivation") OR MAINSUBJECT.EXACT.EXPLODE("Stress") OR "psychological well-being" OR "self concept" OR motivation OR "psychological stress" OR psychological∗ OR self-efficac∗ OR kinesiophobia∗ OR confidence OR anxiety∗ OR stress∗ OR "emotional wellbeing" OR "emotional well-being" OR "self perception" OR "self esteem" OR depress∗ OR depression OR anxiety∗ OR nervous∗ OR fear∗ OR MAINSUBJECT.EXACT.EXPLODE("Physical Activity") OR exercise∗ OR "physical activity") AND (MAINSUBJECT.EXACT.EXPLODE("Quality of Life") OR MAINSUBJECT.EXACT.EXPLODE("Sleep Wake Disorders") OR MAINSUBJECT.EXACT.EXPLODE("Pain") OR MAINSUBJECT.EXACT.EXPLODE("Range of Motion") OR "quality of life" OR sleep OR insomnia∗ OR pain OR "muscle strength" OR strength OR "range of motion" OR flexibility) Limit to peer reviewed
SPORTDiscus:	(DE "Rotator Cuff" OR DE "Shoulder Injuries" OR rotator-cuff∗ OR teres minor OR subscapularis OR infraspinatus OR supraspinatus) AND (DE "WELL-being" OR DE "SELF-perception" OR DE "BODY image" OR DE "SELF-esteem" OR DE "MOTIVATION (Psychology)" OR DE "ACHIEVEMENT motivation" OR DE "INTRINSIC motivation" OR DE "LEVEL of aspiration" OR DE "PSYCHOLOGICAL stress" OR DE "ANXIETY" OR DE "IMMOBILIZATION stress" OR DE "POST-traumatic stress disorder" OR DE "PSYCHOLOGICAL burnout" OR DE "TIME pressure" OR DE "EXERCISE" OR DE "ABDOMINAL exercises" OR DE "AEROBIC exercises" OR DE "ANAEROBIC exercises" OR DE "AQUATIC exercises" OR DE "ARM exercises" OR DE "BACK exercises" OR DE "BREATHING exercises" OR DE "BREEMA" OR DE "BUTTOCKS exercises" OR DE "CALISTHENICS" OR DE "CHAIR exercises" OR DE "CHEST exercises" OR DE "CIRCUIT training" OR DE "COMPOUND exercises" OR DE "COOLDOWN" OR DE "DO-in" OR DE "EXERCISE adherence" OR DE "EXERCISE for children" OR DE "EXERCISE for girls" OR DE "EXERCISE for men" OR DE "EXERCISE for middle-aged persons" OR DE "EXERCISE for older people" OR DE "EXERCISE for people with disabilities" OR DE "EXERCISE for women" OR DE "EXERCISE for youth" OR DE "EXERCISE therapy" OR DE "EXERCISE video games" OR DE "FACIAL exercises" OR DE "FALUN gong exercises" OR DE "FOOT exercises" OR DE "GYMNASTICS" OR DE "HAND exercises" OR DE "HATHA yoga" OR DE "HIP exercises" OR DE "ISOKINETIC exercise" OR DE "ISOLATION exercises" OR DE "ISOMETRIC exercise" OR DE "ISOTONIC exercise" OR DE "KNEE exercises" OR DE "LEG exercises" OR DE "LIANGONG" OR DE "METABOLIC equivalent" OR DE "MULAN quan" OR DE "MUSCLE strength" OR DE "PILATES method" OR DE "PLYOMETRICS" OR DE "QI gong" OR DE "REDUCING exercises" OR DE "RUNNING" OR DE "SCHOOL exercises & recreations" OR DE "SEXUAL exercises" OR DE "SHOULDER exercises" OR DE "STRENGTH training" OR DE "STRESS management exercises" OR DE "TAI chi" OR DE "TREADMILL exercise" OR DE "WHEELCHAIR workouts" OR DE "YOGA" OR DE "PHYSICAL activity" OR psychological∗ OR self-efficac∗ OR kinesiophobia∗ OR confidence OR anxiety∗ OR stress∗ OR "emotional wellbeing" OR "emotional well-being" OR "self perception" OR "self esteem" OR depress∗ OR depression OR anxiety∗ OR nervous∗ OR fear∗ OR exercis∗ OR physical-activit∗) AND (DE "QUALITY of life" OR DE "SLEEP deprivation" OR DE "SLEEP deprivation & health" OR DE "SLEEP disorders" OR DE "MUSCLE strength" OR DE "GRIP strength" OR DE "RANGE of motion of joints" OR DE "JOINT hypermobility" OR DE "JOINT instability" OR DE "JOINT stiffness" OR sleep OR insomnia∗ OR pain OR "muscle strength" OR strength OR "range of motion" OR flexibility) Limit to peer reviewed

We performed a comprehensive search to capture all possible published studies reporting psychological factors and/or PA before and after RC repair surgery and their impact on function/strength, pain, and/or QoL as outcomes. We searched 5 databases (PubMed, Embase, Scopus, SPORTDiscus, and PsycINFO) on August 5, 2025 (searched from inception of the database) to identify potential studies. Furthermore, a hand search using reference lists of all included studies as well as relevant review studies was conducted to identify any additional studies that were not captured during our database searches.

### Study screening/inclusion and charting the data

We used an online tool that is designed specifically for screening articles for review studies (Covidence: https://www.covidence.org). Two authors (H.A. and N.A.) independently screened titles/abstracts and then performed the full-text screening. Conflicts between the 2 authors were resolved by discussion.^27^ A third author was consulted (M.I.) in case an agreement was not reached by the 2 reviewers.

Two authors (H.A. and N.A.) independently prepared the initial data charting form and extracted data from one included study to pilot-test the charting process. The authors then met to discuss any differences and to reach consensus on the data items to be extracted. Based on this agreement, the data extraction sheet was refined to include the authors' names, study year of publication, sample size and demographic data, aims of the study, psychological factors and PA assessments used, and the study's outcomes (when included and/or relevant). The finalized data extraction sheet was subsequently used to extract data from all included studies, and the extracted data were reviewed for consistency prior to reporting the findings of this scoping review.

### Quality assessment for included studies

Study quality assessment was performed for all included studies using the Joanna Briggs Institute Critical Appraisal Checklist for cohort studies.^28^ One author (H.A.) independently assessed each study's quality and reviewed its results with another author (N.A.) for the rate of each criterion. The same 2 authors resolved any disagreements (if any) via discussion. A score of one was assigned to each criterion rated as “yes” and zero to items rated as “no.” When there was insufficient data in the study to determine a rating score, “not applicable” was assigned.^28^^,^^43^ For each study quality assessment, scores were converted into a percentage score, ranging from 0 (indicates the lowest score) and 100 (indicates the highest score) to enable easier comparisons. The percentage was calculated based on a reduced number of overall criteria in the case that a “not applicable” designation was assigned for a given item. Studies were identified as “low” quality if the study was assigned a score of 0–24%, “low–moderate” if the study was assigned a score of 25–49%, “moderate–high” if the study was assigned a score of 50–74%, and “high” quality if the study was assigned a score of 75–100%^28^^,^^43^ (Table III).

**Table III:** Study quality assessment.

Authors and year of publication	1. Were the two groups similar and recruited from the same population?	2. Were the exposures measured similarly to assign people to both exposed and unexposed groups?	3. Was the exposure measured in a valid and reliable way?	4. Were confounding factors identified?	5. Were strategies to deal with confounding factors stated?	6. Were the groups/participants free of the outcome at the start of the study (or at the moment of exposure)?	7. Were the outcomes measured in a valid and reliable way?	8. Was the follow-up time reported and sufficient to be long enough for outcomes to occur?	9. Was follow-up complete, and if not, were the reasons to loss to follow-up described and explored?	10. Were strategies to address incomplete follow-up utilized?	11. Was appropriate statistical analysis used?	# Criteria	Total	Score
Deniz et al (2022)^17^	Yes	Yes	Yes	Yes	No	Yes	Yes	Yes	Yes	No	Yes	9/11	81.80%	High
Kuechly et al (2024)^40^	Not applicable	Not applicable	Yes	Yes	No	Yes	Yes	Yes	Yes	No	Yes	7/9 (2 N/A)	77.80%	High
Longo et al (2023)^43^	Not applicable	Not applicable	Yes	Yes	No	Yes	Yes	Yes	Yes	No	Yes	7/9 (2 N/A)	77.80%	High
Cho et al (2015)^10^	Not applicable	Not applicable	Yes	Yes	No	Yes	Yes	Yes	Yes	No	Yes	7/9 (2 N/A)	77.80%	High
Kim et al (2024)^37^	Yes	Yes	Yes	Yes	Yes	Yes	Yes	Yes	No	No	Yes	9/11	81.80%	High
Wang et al (2022)^75^	Yes	Yes	Yes	Yes	No	Yes	Yes	Yes	No	No	Yes	8/11	72.70%	Moderate-high
Potter et al (2015)^61^	Yes	Yes	Yes	Yes	Yes	Yes	Yes	Yes	Yes	No	Yes	10/11	91.90%	High
Singh et al (2018)^69^	Yes	Yes	Yes	Yes	No	Yes	Yes	Yes	Yes	No	Yes	9/11	81.80%	High
Hessburg et al (2021)^29^	Yes	Yes	Yes	Yes	No	Yes	Yes	Yes	No	No	Yes	8/11	72.70%	Moderate-high
Sabo et al (2023)^65^	Not applicable	Not applicable	Yes	Yes	No	Yes	Yes	Yes	Yes	Yes	Yes	8/9 (2 N/A)	88.90%	High
Feltri et al (2024)	No	No	Yes	Yes	Yes	Yes	Yes	Yes	Yes	Yes	Yes	9/11	81.80%	High
Chu et al (2021)^13^	Yes	Yes	Yes	Yes	No	Yes	Yes	Yes	Yes	Yes	Yes	10/11	91.90%	High
Shaikh et al (2023)^68^	Yes	Yes	Yes	Yes	Yes	Yes	Yes	Yes	Yes	Yes	Yes	11/11	100.00%	High

### Synthesis of results

A synthesis and thematic analysis of included studies was performed to facilitate understanding of the breadth and scope of the current evidence regarding psychological factors and PA before and after RC repair surgery and their impact on function, strength, and pain. A variety of factors were identified; however, the consistency with which these factors were examined varied substantially. Some factors were repeatedly explored, whereas others received limited attention or were entirely absent from the existing literature. Thereby, a comprehensive summary was generated, and the findings were divided into 3 different themes based on the reported outcome measures before and after RC repair surgery and the associations between them. These themes included 1) frequently explored variables, 2) under-investigated variables, and 3) associations between identified psychological factors, function, strength, and pain.

## Results

### Search results

**Figure 1:**
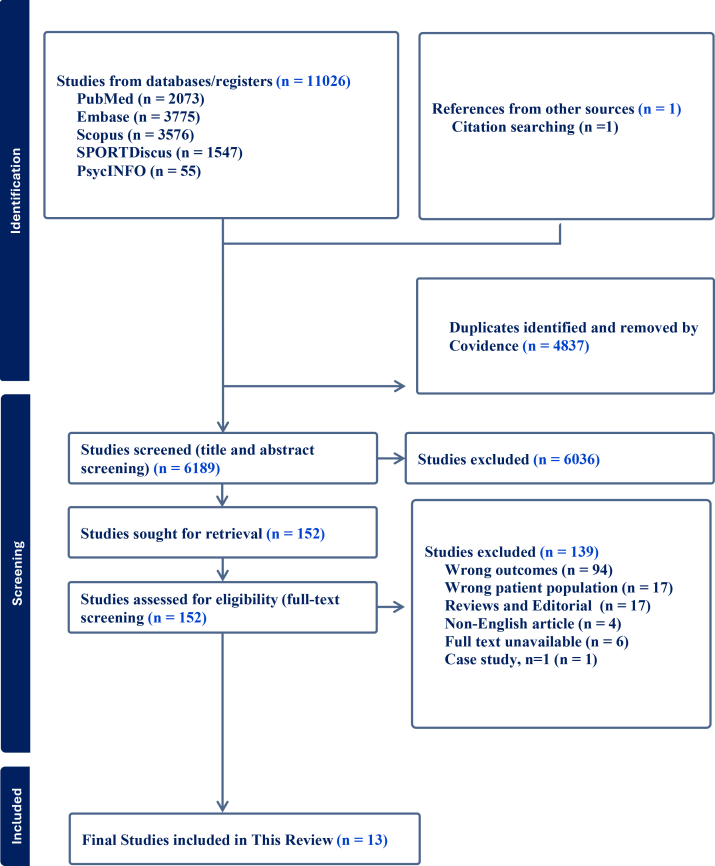
PRISMA flow diagram. *PRISMA*, Preferred Reporting Items for Systematic Reviews and Meta-Analyses.

Our database and hand search yielded 11,027 potential studies. These studies were found in: PubMed (n = 2,073), Embase (n = 3,775), Scopus (n = 3,576), SPORTDiscus (n = 1,547), PsycINFO (n = 55), and via hand searches (n = 1) (Fig. 1). Of these, 4,837 were duplicates and were removed (identified by Covidence), and the remaining studies' (n = 6,189) titles and abstracts were screened. Following abstract/title screening, 6,036 were excluded. We reviewed 152 potential full-texts for inclusion, and eventually 13 studies met our inclusion and exclusion criteria (Fig. 1). Excluded full-text studies (n = 140) were excluded primarily due to not including psychological factors or PA measures (n = 94), not including those with RC pathology before/after repair (n = 18), review or editorial articles (n = 17), full-texts being unavailable (n = 6), non-English studies (n = 4), or case studies (n = 1). Of the included studies (n = 13), 4 were conducted in the United States,^27^^,^^37^^,^^58^^,^^65^ 2 in Canada,^62^^,^^66^ 2 in South Korea,^10^^,^^34^ 2 in China,^13^^,^^73^ 1 in Turkey,^17^ 1 in Italy,^41^ and 1 in Switzerland.^20^ Out of the studies included (n = 13), 3 were retrospective and 10 were prospective studies, and all of them were published in 2015 and later.

### Patient characteristics

Across all included studies (n = 13), there were 2,425 total included patients (55% males). The mean age of patients across all studies was 59.6 (±9.9) years. Out of the 13 studies included, 11 reported arthroscopic RC repair surgery as their intervention.^13^^,^^17^^,^^20^^,^^27^^,^^34^^,^^37^^,^^41^^,^^58^^,^^62^^,^^65^^,^^66^ One reported arthroscopic RC repair surgery was used for 79% of their patients, and 21% of patients were treated by mini-open RC repair.^10^ Another one reported using a mini-open RC repair surgery with their patients.^73^ Follow-up periods varied among the studies included. Most studies collected outcome measures before and 6 months after surgery (n = 8),^10^^,^^17^^,^^20^^,^^27^^,^^37^^,^^41^^,^^65^^,^^66^ while some of studies collected their outcome measures before and followed participants for up to one year post-operatively (n = 6).^10^^,^^20^^,^^27^^,^^58^^,^^62^^,^^73^ One study collected outcome measures before and at 2 years after surgery,^13^ while another study collected outcome measures before and at 2 years or later after surgery.^34^ Shorter follow-up periods were reported often, as one study collected outcome measures 3 months after repair surgery,^10^ another study considered 6 weeks as a follow-up period,^73^ and the shortest follow-up period reported was one month after repair surgery.^41^

Of the included studies, 10 studies collected data on depression symptoms as a psychological factor in patients with RC pathology,^10^^,^^13^^,^^17^^,^^20^^,^^27^^,^^34^^,^^41^^,^^62^^,^^65^^,^^66^ 7 studies collected data on anxiety symptoms,^10^^,^^13^^,^^20^^,^^34^^,^^41^^,^^62^^,^^66^ 2 studies collected data on kinesiophobia,^17^^,^^73^ 2 studies collected data related to the negative pain thoughts.^37^^,^^69^We found no studies that collected any PA-related data in patients with RC pathology before and after RC repair surgery.

Critical appraisal scores for all included studies ranged from 72.7 to 100.^10^^,^^13^^,^^17^^,^^20^^,^^27^^,^^34^^,^^37^^,^^41^^,^^58^^,^^62^^,^^65^^,^^66^^,^^73^ Most of the studies included (11 out of 13) were categorized in the high-quality range (77.8-100).^10^^,^^13^^,^^17^^,^^17^^,^^20^^,^^34^^,^^37^^,^^41^^,^^58^^,^^62^^,^^66^ Two of the included studies were found to be of moderate-high quality, with an average score of 72.7^27^^,^^73^ (Table III).

### Measures of psychological well-being before and after rotator cuff repair

#### Theme 1: frequently-explored variables (depression, anxiety, and distress)

Out of the 13 included studies, 10 studies collected data on depression symptoms^10^^,^^13^^,^^17^^,^^20^^,^^27^^,^^34^^,^^41^^,^^62^^,^^65^^,^^66^ and 7 of these 10 also collected data on anxiety symptoms (n = 1,926 total patients).^10^^,^^13^^,^^34^^,^^41^^,^^62^^,^^66^ Depression level was measured using the Hospital Anxiety and Depression Scale (HADS)^80^ (n = 6),^10^^,^^13^^,^^34^^,^^41^^,^^62^^,^^66^ the Patient-Reported Outcomes Measurement Information System (PROMIS)^8^ (n = 3),^20^^,^^27^^,^^65^ and the Beck Depression Inventory (BDI)^61^ (n = 1).^17^ All studies found that measures of depression significantly improved 6 months^10^^,^^17^^,^^20^^,^^27^^,^^41^^,^^65^^,^^66^ or 1 year following RC repair compared to before surgery^10^^,^^13^^,^^20^^,^^27^^,^^34^^,^^62^ (Table IV). All studies that collected data on anxiety (n = 7) used the HADS.^10^^,^^13^^,^^34^^,^^41^^,^^62^^,^^66^^,^^80^ Longo and colleagues^41^ found that depression and anxiety level were significantly improved 1 month, 3 months, and 6 months after RC repair surgery compared to before surgery (*P* < 01).^41^ In addition to depression and anxiety symptoms, Potter and colleagues^58^ also assessed psychological distress, using the Distrdsess Risk Assessment Method questionnaire (sample size = 85), and found that the prevalence of psychological distress decreased from 26 of 85 (41%) pre-operatively to 14 of 70 (20%) one year after RC repair. However, when comparing distressed patients with nondistressed patients, there was no significant difference in post-operative visual analog scale (VAS) for pain, Simple Shoulder Test, or American Shoulder and Elbow Surgeons (ASES) 1 year following arthroscopic RC repair^58^ (Table IV).

**Table IV:** Outcome measures reported before and after RC repair surgery.

Authors and publication year	Demographic	Outcome measures	Before RC repair		At follow-up	*P* value
Deniz et al (2022)^17^	(n = 45) (53.3% male; 24). Age 57.78 ± 6.46	Beck Depression Inventory (BDI)	14 ± 10–23		(6 mo) 9 ± 6–15	.001∗∗
		Tampa Scale of Kinesiophobia (TSK)	55 ± 52–58		(6 mo) 24 ± 21–34	.001∗∗
		Shoulder Pain and Disability Index (SPADI)	83.07 ± 69.23–93.07		(6 mo) 13.84 ± 11.53–26.15	.001∗∗
		Constant Murley score (CMS)	24 ± 16–53		(6 mo) 86 ± 73–91	.001∗∗
Kuechly et al (2024)^40^	(n = 74) (56% males; 42). Age 56.49 ± 9.98	American Shoulder and Elbow Surgeons (ASES)	38.8 ± 19.4		(6 mo) 75.4 ± 19.4	<.001∗∗
		Short Form Health Survey (SF-12) physical health score	35.5 ± 7.6		(6 mo) 43.6 ± 9.9	<.001∗∗
		Short Form Health Survey (SF-12) mental health score	52.2 ± 11.0		(6 mo) 53.5 ± 9.3	.37
		Negative Pain Thoughts Questionnaire (NPTQ-SF)	12.8 ± 4.9		(6 mo) 8.5 ± 4.6	<.001∗∗
Longo et al (2023)^43^	(n = 43) (51% males; 22). Age 63.3 ± 11.1 yr	The Hospital Anxiety and Depression Scale (HADS)-Anxiety	6.7 ± 8.7		(1 mo) 5.4 ± 3.8	<.001∗∗
					(3 mo) 3.6 ± 3.4	<.001∗∗
					(6 mo) 3.2 ± 3.5	<.001∗∗
		The Hospital Anxiety and Depression Scale (HADS)-Depression	2.2 ± 3.7		(1 mo) 3.8 ± 4.3	<.001∗∗
					(3 mo) 2.5 ± 3.7	<.001∗∗
					(6 mo) 2.2 ± 3.7	<.001∗∗
		Constant Murley score (CMS) score	41.1 ± 16.7		(1 mo) 31.9 ± 10.3	<.001∗∗
					(3 mo) 57.6 ± 12.9	<.001∗∗
					(6 mo) 64.3 ± 9.9	<.001∗∗
		Short Form Survey (SF-36)	99.6 ± 8.5		(1 mo) 97.6 ± 7.7	<.001∗∗
					(3 mo) 102.7 ± 7.5	<.001∗∗
					(6 mo) 103.3 ± 6.9	<.001∗∗
Kim et al (2024)^37^	(n = 173) (30.1% male; 52). Age 67.3 ± 7.7 yr	Visual Analog Scale (VAS)	Young age group (54–69 yr)	5.58 ± 1.95	(2.2 to 6.3 yr) 1.53 ± 1.39	.006∗
			Old age group (70–89 yr)	5.64 ± 2.12	(3.8 ± 2.23 yr) 1.18 ± 1.34	.005∗
		Constant Murley score (CMS) score	Young age group (54–69 yr)	77.81 ± 13.75	(2.2 to 6.3 yr) 77.81 ± 13.75	.001∗∗
			Old age group (70–89 yr)	51.65 ± 10.66	(3.8 ± 2.23 yr) 70.17 ± 12.82	.001∗∗
		The Hospital Anxiety and Depression Scale (HADS)-Anxiety	Young age group (54–69 yr)	8.85 ± 3.05	(2.2 to 6.3 yr) 7.48 ± 0.93	.041∗
			Old age group (70–89 yr)	9.64 ± 3.78	(3.8 ± 2.23 yr) 6.65 ± 0.77	.006∗
		The Hospital Anxiety and Depression Scale (HADS)-Depression	Young age group (54–69 yr)	8.42 ± 2.71	(2.2 to 6.3 yr) 7.37 ± 0.59	.033∗
			Old age group (70–89 yr)	9.22 ± 3.36	(3.8 ± 2.23 yr) 6.87 ± 0.96	.013∗
		Shoulder Activity Scale (SAS)	Young age group (54–69 yr)	7.11 ± 3.05	(2.2 to 6.3 yr) 14.91 ± 4.43	.003∗
			Old age group (70–89 yr)	5.31 ± 2.14	(3.8 ± 2.23 yr) 11.38 ± 4.02	.021∗
		Short Form Health Survey (SF-12) physical health score	Young age group (54–69 yr)	33.16 ± 8.77	(2.2 to 6.3 yr) 52.19 ± 13.65	<.001∗∗
			Old age group (70–89 yr)	28.34 ± 7.11	(3.8 ± 2.23 yr) 40.15 ± 10.59	.016∗
		Short Form Health Survey (SF-12) mental health score	Young age group (54–69 yr)	55.18 ± 12.33	(2.2 to 6.3 yr) 61.85 ± 15.63	.045∗
			Old age group (70–89 yr)	52.46 ± 11.71	(3.8 ± 2.23 yr) 60.32 ± 14.19	.037∗
Singh et al (2018)^69^	(n = 64) (69% male; 44). Age 50 ± 8 yr	The Quick Disabilities of the Arm, Shoulder, and Hand (QuickDASH)	50 ± 21		(6 mo) 46 ± 21	>.05
		The Hospital Anxiety and Depression Scale (HADS)-Anxiety	Normal 58%, borderline abnormal 22%, abnormal 20%		(6 mo) normal 69%, borderline abnormal 11%, abnormal 16%	>.06
		The Hospital Anxiety and Depression Scale (HADS)- Depression	Normal 66%, borderline abnormal 14%, abnormal 20%		(6 mo) normal 73%, borderline abnormal 11%, abnormal 16%	>.07
Hessburg et al (2021)^29^	(n = 340) (54.1% male; 184). 59.4 ± 9.1 yr	Patient-Reported Outcomes Measurement Information System (PROMIS)- Depression	Number of participants with clinical depression 61.5		(6 mo) 51.1	Improvement −8.9, *P* < .001
					(12 mo) 52.6	
			Number of participants without clinical depression	44.7	(6 mo) 41.9	Improvement 15.4, *P* < .001
					(12 mo) 41.4	
		Patient-Reported Outcomes Measurement Information System (PROMIS)- upper extremity physical function	Total cohort	30.0 (14.7-45.9)	(6 mo) 38.0 (14.7-61.0)	Improvement 15.4, *P* < .001
					(12 mo) 45.4 (22.3-61.0)	
			Number of participants with clinical depression	26.7 (14.7-43.1)	(6 mo) 35.5 (17.5-61.0)	Improvement 12.2, *P* < .001
					(12 mo) 38.9 (22.3-61.0)	
			Number of participants without clinical depression	30.8 (14.7-45.9)	(6 mo) 38.6 (14.7-61.0)	Improvement 16.1, *P* < .001
					(12 mo) 46.9 (26.3-61.0)	
Sabo et al (2023)^65^	(n = 148) (48.6% male; 72). Age 55.1 ± 8.3 yr	Pain Catastrophizing Score (PCS)	______		No significant differences in change of PSC	.6
		The Hospital Anxiety and Depression Scale (HADS)-Anxiety	______		(12 mo) single trajectory found a gradual decline in anxiety symptom scores over time	______
		The Hospital Anxiety and Depression Scale (HADS)-Depression	______		(12 mo) stable (less-favorable) trajectory included a history of cardiovascular disease, current smoking, higher baseline pain scores, and worse baseline WORC, HADS-D, HADS-A, and PCS scores	______
Feltri et al (2025)^21^	(n = 973) (62.8% male; 611). Age 57.3 ± 9.4 yr	Constant and Murley score (CMS)	50.4 ± 18.3		(6 mo) 68.4 ± 15.2	<.001∗∗
					(12 mo) 77.2 ± 12.1	<.001∗∗
		Oxford Shoulder Score (OSS)	27.3 ± 8.9		(6 mo) 39.8 ± 7.6	<.001∗∗
					(12 mo) 43.3 ± 6.7	<.001∗∗
		Patient-Reported Outcomes Measurement Information System (PROMIS)- Anxiety	50.1 ± 8.9		(6 mo) 45.9 ± 8.0	<.001∗∗
					(12 mo) 45.0 ± 7.5	<.001∗∗
		Patient-Reported Outcomes Measurement Information System (PROMIS)- Depression	51.4 ± 8.8		(6 mo) 46.6 ± 7.9	<.001∗∗
					(12 mo) 45.4 ± 7.3	<.001∗∗
Chu et al (2021)^13^	(n = 93) (43.0% male; 40). Age 54.8 ± 8.1 yr	Hospital Anxiety and Depression Scale (HADS)- Anxiety	Staged ARCR	Normal 26 ± 61.9, abnormal 16 ± 38.1, mild 7 ± 16.7, moderate 6 ± 14.3, and severe 3 ± 7.1	(36 to 60 mo) normal 39 ± 92.9, abnormal 3 ± 7.1, mild 7.1, moderate 0, and severe 0	.003∗
			Simultaneous ARCR	Normal 31 ± 60.8, abnormal 20 ± 39.2, mild 9 ± 21.4, moderate 6 ± 14.3, and severe 5 ± 9.8	(25-59 mo) normal 47 ± 92.2, abnormal 4 ± 7.8, mild 2 ± 3.9, moderate 2 ± 3.9, and severe 0	<.001∗∗
		The Hospital Anxiety and Depression Scale (HADS)-Depression	Staged ARCR	Normal 33 ± 78.6, abnormal 9 ± 21.4, mild 6 ± 14.3, moderate 2 ± 4.8, and severe 1 ± 2.4	(36 to 60 mo) normal 39 ± 92.9, abnormal 3 ± 7.1, mild 7.1, moderate 0, and severe 0	0.024∗
			Simultaneous ARCR	Normal 38 ± 74.5, abnormal 13 ± 25.5, mild 5 ± 9.8, moderate 5 ± 9.8, & Severe 3 ± 5.9	(25-59 mo) normal 47 ± 92.2, abnormal 4 ± 7.8, mild 2 ± 3.9, moderate 1 ± 2.0, and severe 1 ± 2.0	.005∗
		Pittsburgh Sleep Quality Index (PSQI)	Staged ARCR	∗∗∗	(24 mo) ∗∗∗	<.05∗
			Simultaneous ARCR	∗∗∗	(24 mo) ∗∗∗	<.05∗
		World Health Organization Quality of Life Scale–Abbreviated Version (WHOQOL-BREF)	Staged ARCR	∗∗∗	(24 mo) ∗∗∗	<.05∗
			Simultaneous ARCR	∗∗∗	(24 mo) ∗∗∗	<.05∗
		Constant and Murley score (CMS)	Staged ARCR	∗∗∗	(24 mo)∗∗∗	<.05∗
			Simultaneous ARCR	∗∗∗	(24 mo)∗∗∗	<.05∗
		American Shoulder and Elbow Surgeons (ASES)	Staged ARCR	∗∗∗	(24 mo)∗∗∗	<.05∗
			Simultaneous ARCR	∗∗∗	(24 mo)∗∗∗	<.05∗
Shaikh et al (2023)^68^	(n = 306) (52.3% male; 160). Age 60.3 ± 8.4	Patient-Reported Outcomes Measurement Information System (PROMIS)- Depression	ADILow	43.3 ± 13.4	(6 mo) 36.6 ± 18.2	__
			ADIHigh	44.8 ± 16.2	(6 mo) 40.9 ± 18.3	__
Potter et al (2015)^61^	(n = 70) (74.3% male; 52). Age 61 ± 62 yr.	Distress Risk Assessment Method questionnaire	Number of participants in the normal group	44	(12 mo) 56	__
			Number of participants in the distressed group	26	(12 mo) 14	__
Cho et al (2015)^10^	(n = 47) (43% male; 20). Age 57 ± 8 yr.	Hospital Anxiety and Depression Scale (HADS)- Anxiety	4.3 ± 4.3		(3 mo) 2.5 ± 4.2	<.001∗∗
					(6 mo) 2.1 ± 2.9	<.001∗∗
					(12 mo) 1.4 ± 2.4	<.001∗∗
		The Hospital Anxiety and Depression Scale (HADS)-Depression	3.7 ± 3.3		(3 mo) 2.4 ± 3.2	.148
					(6 mo) 2.4 ± 2.5	.148
					(12 mo) 2.1 ± 2.3	.003∗
		Pittsburgh Sleep Quality Index (PSQI) score	6.6 ± 3.6		(12 mo) 4.2 ± 3.3	<.001∗∗
		WHO Quality of Life-BREF (WHOQOL-BREF) score	60.4 ± 11.0		(12 mo) 67.4 ± 11.8	<.001∗∗
Wang et al (2022)^75^	(n = 49) (61.2% male; 30). Age 53.7 ± 8.9 yr	Oxford Shoulder Score (OSS)	Trial group - high kinesiophobia (TSK >37) 52.54 ± 1.30	52.54 ± 1.30	(6 weeks) 32.96 ± 1.48	__
					(12 mo) 27.42 ± 1.47	__
			Control group with - low kinesiophobia (TSK ≤37) 33.43 ± 1.67	33.43 ± 1.67	(6 weeks) 28.17 ± 1.03	__
					(12 mo) 26.87 ± 1.18	__

*RC*, rotator cuff; *WORC*, Western Ontario Rotator Cuff; *ARCR*, arthroscopic rotator cuff repair; *PCS*, Pain Catastrophizing Scale; *ADI*, area deprivation index; ADIHigh, area deprivation index high; *ADILow*, area deprivation index low.

Significance levels are indicated as follows: ∗*P* < .05; ∗∗*P* < .01; ∗∗∗*P* < .001.

#### Theme 2: under-investigated variables (negative pain thoughts and kinesiophobia)

Negative pain-related thoughts were examined in one study by Kuechly and colleagues^37^ using the Negative Pain Thoughts Questionnaire-short form (NPTQ-SF),^16^ where a score of ≥8 is considered clinically significant for indicating a high level of negative pain-related thoughts.^16^ This study (sample size = 74) found that patients with RC pathology experienced negative pain-related thoughts, but that they significantly decreased 6 months after RC repair surgery (pre-operative NPTQ-SF = 12.8 ± 4.9; post-operative NPTQ-SF = 8.5 ± 4.6) (Table IV). In a study by Sabo and colleagues^62^ (sample size = 148), pain catastrophizing was measured using the Pain Catastrophizing Score (PCS),^70^ finding no significant change in PCS scores before to one year after RC repair surgery.

Of the 13 included studies, 2 studies collected data on kinesiophobia before and after RC repair.^17^^,^^73^ The Tampa Scale of Kinesiophobia^48^ was used in both of the studies; Deniz and colleagues^17^ reported a significant reduction in kinesiophobia 6 months after RC repair surgery compared to before surgery. Wang and colleagues^73^ reported a serial reduction of the number of participants in the high kinesiophobia group 6 weeks and 12 months after repair surgery (52, 32, and 27 participants, respectively) (Table IV).

#### Theme 3: reported associations between identified psychological well-being measures and surgery outcomes

##### Depression and anxiety with shoulder function and quality of life

Longo and colleagues^41^ reported an association between lower (better) pre-operative depression and anxiety scores (HADS-A and HADS-D, respectively) with higher (better) pre-operative shoulder function and QoL as measured by Constant Murley score^15^ and Short Form Health Survey (SF)-36, respectively.^76^ The same associations were reported in the post-operative follow-up at 3 months and 6 months after RC repair surgery^41^ (Table V). Singh and colleagues^66^ similarly found a significant strong correlation between higher 6-month post-operative depression scores (measured by HADS-D) and lack of intention to engage in return to work behavior measured by the Readiness for Return to Work^21^ scale 6 months after RC repair surgery (Table V).

**Table V:** Associations between psychological well-being measures and RC repair outcomes.

No.	Authors and year of publication	Associations between measures	Association values
1	Longo et al (2023)^43^	Pre-operative anxiety (HADS-A) and pre-operative (CMS)	*r* = −0.3, *P* = .032∗
		Pre-operative anxiety (HADS-A) and pre-operative (SF-36)	*r* = −0.6, *P* < .001∗
		Pre-operative depression (HADS-D) and pre-operative (CMS)	*r* = −0.5, *P* = .001∗
		Pre-operative depression (HADS-D) and pre-operative (SF-36)	*r* = −0.6, *P* < .001∗
		6 mo post-operative anxiety (HADS-A) and 6 mo post-operative (CMS)	*r* = −0.4, *P* < .001∗
		6 mo post-operative anxiety (HADS-A) and 6 mo post-operative (SF-36)	*r* = −0.6, *P* < .001∗
		6 mo post-operative depression (HADS-D) and 6 mo post-operative (CMS)	*r* = −0.5, *P* = .002∗
		6 mo post-operative depression (HADS-D) and 6 mo post-operative (SF-36)	*r* = −0.5, *P* < .001∗
2	Singh et al (2018)^69^	6 mo post-operative depression (HADS-D) and 6 mo post-operative readiness for return-to-work scale (RRTW)	*r* = 0.64, *P* = .001∗
3	Sabo et al (2023)^65^	Pre-operative anxiety (HADS-A) and 1 yr post-operative (WORC)	*r* = −0.43, *P* > .0001∗
		Pre-operative depression (HADS-D) and 1 yr post-operative (WORC)	*r* = −0.42, *P* < .0001∗
		1 yr post-operative anxiety (HADS-A) and post-operative anxiety (HADS-A)	*r* = −0.69, *P* > .0001∗
		1 yr post-operative depression (HADS-D) and 1 yr post-operative (WORC)	*r* = −0.61, *P* > .0001∗
		1 yr non-improving depression (HADS-D) and pre-operative pain (VAS)	OR = 1.09, 95% CI [1.01–1.16]∗
		1 yr non-improving depression (HADS-D) and pre-operative (PCS)	OR = 1.06, 95% CI [1.03–1.09]∗
		1 yr non-improving depression (HADS-D) and pre-operative depression (HADS-D)	OR = 1.34, 95% CI [1.20–1.49]∗
		1 yr non-improving depression (HADS-D) and pre-operative anxiety (HADS-A)	OR = 1.25, 95% CI [1.13–1.38]∗
		Pre-operative (PCS) and 1 yr post-operative (WORC)	*r* = −0.38, *P* < .0001∗
		1 yr post-operative (PCS) and 1 yr post-operative (WORC)	*r* = −0.60, *P* < .0001∗
4	Cho et al (2013)^10^	Pre-operative anxiety (HADS-A) and 1 yr pain (VAS)	*r* = 0.115, *P* = .515
		Pre-operative anxiety (HADS-A) and 1 yr pain (UCLA)	*r* = −0.089, *P* = .657
		Pre-operative anxiety (HADS-A) and 1 y (ASES)	*r* = −0.624, *P* = .335
		Pre-operative depression (HADS-D) and 1 yr pain (VAS)	*r* = −0.073, *P* = .515
		Pre-operative depression (HADS-D) and 1 yr pain (UCLA)	*r* = −0.027, *P* = .920
		Pre-operative depression (HADS-D) and 1 yr (ASES)	*r* = 0.235, *P* = .785
5	Kuechly et al (2024)^40^	Pre-operative (NPTQ-SF) and pre-operative (SF-12 PHS)	*r* = −0.285, *P* = .014∗
		Pre-operative (NPTQ-SF) and pre-operative (SF-12 MHS)	*r* = −0.421, *P* < .001∗
		Pre-operative (NPTQ-SF) and pre-operative (ASES)	*r* = −0.199, *P* = .089
		Pre-operative (NPTQ-SF) and 6 mo post-operative (SF-12 PHS)	*r* = −0.203, *P* = .082
		Pre-operative (NPTQ-SF) and 6 mo post-operative (SF-12 MHS)	*r* = −0.113, *P* = .337
		Pre-operative (NPTQ-SF) and 6 mo post-operative (ASES)	*r* = −0. 043, *P* = .713
		6 mo post-operative (NPTQ-SF) and pre-operative (SF-12 PHS)	*r* = −0.122, *P* = .3
		6 mo post-operative (NPTQ-SF) and pre-operative (SF-12 MHS)	*r* = −0.305, *P* = .008∗
		6 mo post-operative (NPTQ-SF) and pre-operative (ASES)	*r* = 0.074, *P* = .531
		6 mo post-operative (NPTQ-SF) and 6 mo post-operative (SF-12 PHS)	*r* = −0.727, *P* < .001∗
		6 mo post-operative (NPTQ-SF) and 6 mo post-operative (SF-12 MHS)	*r* = −0.331, *P* = .004∗
		6 mo post-operative (NPTQ-SF) and 6 mo post-operative (ASES)	*r* = – 0.709, *P* < .001∗
		6 mo post-operative (NPTQ-SF) and 6 mo post-operative (ASES MCID)	*r* = – 0.298, *P* < .009∗
		6 mo post-operative (NPTQ-SF) and 6 mo post-operative (ASES PASS)	*r* = – 0.548, *P* < .001∗
		6 mo post-operative (NPTQ-SF) and 6 mo post-operative (Satisfaction)	*r* = – 0. 473, *P* < .001∗
		6 mo post-operative (NPTQ-SF) and 6 mo post-operative (VAS Pain)	*r* = 0. 599, *P* < .001∗

*HADS*, Hospital Anxiety and Depression Scale; *ASES*, American Shoulder and Elbow Surgeons; *VAS*, visual analog scale; *PASS*, Patient Acceptable Symptom State; *NPTQ-SF*, Negative Pain Thoughts Questionnaire-short form; *MCID*, Minimal Clinically Important Difference; *UCLA*, University of California-Los Angeles; *CMS*, Constant Murley score; *SF-12*, Short Form Health Survey; *PCS*, Pain Catastrophizing Score; *WORC*, Western Ontario Rotator Cuff; *PHS*, physical health score; *MHS*, mental health score.

∗ Indicates a statistically significant association.

Sabo and colleagues^62^ found that decreased (better) depression and anxiety (HADS-A and HADS-D, respectively) one year after RC repair surgery was correlated with higher (better) Western Ontario Rotator Cuff (WORC)^35^ Index scores, which measures QoL and functional ability in patients with RC pathology (Table V). Moreover, Sabo and colleagues^62^ found that non-improving depression (HADS-D) one year after RC repair surgery was associated with worse pre-operative pain, worse pre-operative function using the WORC, worse pre-operative pain catastrophizing using PCS, worse pre-operative depression level, and worse pre-operative anxiety (Table V).

In contrast, Cho and colleagues^10^ reported weak and nonsignificant correlations between pre-operative anxiety and depression symptoms and shoulder pain or function 12 months after repair surgery (Table V). Specifically, there were weak and nonsignificant associations between HADS-D scores and VAS pain scores, the University of California-Los Angeles shoulder scale scores,^1^ and the ASES^47^ scores 12 months after RC repair surgery.^10^ Similarly, HADS-A scores were not significantly correlated with VAS pain scores, University of California-Los Angeles shoulder scale scores, or ASES scores 12 months after RC repair surgery^10^ (Table V).

##### Negative pain-related thoughts with shoulder function, pain, and quality of life

Kuechly and colleagues^37^ reported that higher (worse) NPTQ-SF pre-operative negative pain-related thoughts were associated with lower (worse) pre-operative physical and mental health-related QoL (measured by SF-12^79^) and lower (worse) shoulder function (measured by ASES) (Table IV). Additionally, those with chronic pain, categorized using International Classification of Diseases, 10th Revision, code G89, had significantly lower pre-operative SF-12 physical health score (PHS) scores (32.7 vs. 36.9) and 6 months post-operative SF-12 PHS scores (40.3 vs. 45.2). Lower (better) 6-month post-operative negative pain-related thought scores were associated with lower pain levels 6 months post-operative (Table V), and lower post-operative negative pain-related thought scores were correlated with achieving both Patient Acceptable Symptom State cutoffs and exceeding the Minimal Clinically Important Difference change on the ASES.^37^ Lastly, lower (better) post-operative negative pain-related thought scores were also correlated with higher (better) post-operative physical and mental health-related QoL (SF-12), shoulder function on the ASES, and patient satisfaction (Table V).^37^

##### Pain catastrophizing with shoulder function

Sabo and colleagues^62^ found that higher (worse) pre-operative pain catastrophizing PCS scores were weakly associated with worse WORC scores one year after surgery, whereas higher (worse) pain catastrophizing scores one year after surgery were moderately associated with lower (worse) WORC scores also at one year after surgery.^62^

## Discussion

Despite a growing body of literature in orthopedics, this scoping review highlights that studies examining psychological well-being and PA and among individuals with RC pathology undergoing repair are limited, with few reporting associations with outcome measures and an overall absence of PA measures. The current scoping review aimed to bridge this gap by synthesizing the existing evidence regarding psychological well-being and PA levels of patients with RC pathology before and after surgical repair. Of the 13 studies included, all reported measures of psychological well-being, whereas none reported PA levels before and after RC repair (remains a critical gap in literature). The included studies reported that depression and anxiety symptoms were consistently present in patients undergoing RC repair surgery. Experiencing less depression and anxiety symptoms before and after RC repair surgery was associated with lower pain, better shoulder function, and overall faster recovery. While fewer studies have explored negative pain-related thoughts, pain catastrophizing, and kinesiophobia in patients with RC pathology before and after surgical repair, these factors were generally associated with decreased shoulder function and worse QoL.

Our findings demonstrate that depression and anxiety symptoms are not only commonly experienced by patients with RC pathology but are also associated with pain intensity, functional recovery, and QoL following RC repair. All the studies included reported an increased level of depression and anxiety symptoms (measured via BDI, HADS, and PROMIS) before RC repair compared to before RC repair.^10^^,^^13^^,^^17^^,^^20^^,^^27^^,^^34^^,^^41^^,^^62^^,^^65^^,^^66^ Our results aligned with a previously published systematic review (n = 15 studies) that found an association between pre-operative psychological factors (ie, depression or anxiety) and the level of disability and pain experienced by patients with RC pathology before repair surgery as well as post-operative outcome measures related to shoulder pain and function.^32^ In addition, our findings were aligned with a systematic review that explored anxiety and depression in patients undergoing other orthopedic procedures, such as lower extremity surgeries.^19^^,^^33^^,^^72^ Longo and colleagues^41^ found that the increased level of pre-operative depression and anxiety predicts worse pain and function following RC repair surgery. Together, these findings reinforce the important role of addressing psychological challenges in patients with RC pathology to promote functional recovery and overall RC repair outcomes. Moreover, our review identified some variation in the reported results related to the association between depression and anxiety with shoulder function and pain.^10^^,^^41^^,^^62^ These variations could be influenced by cross-cultural differences; included studies were conducted across countries around the world including Italy,^41^ South Korea,^10^^,^^34^ and Canada.^62^^,^^66^ Furthermore, differences in outcomes may be explained by variations in rehabilitation protocols,^54^ and/or the level of patient support from family or friends.

In addition to depression and anxiety, pain-related beliefs were infrequently measured/reported before and after RC repair surgery,^37^^,^^62^ despite their clinical significance in other orthopedic conditions.^50^ Negative pain thoughts and pain catastrophizing were reported in only 2 studies in our review.^37^^,^^62^ Experiencing negative pain thoughts before RC repair surgery contributed to worse physical and psychological outcomes as well as worse QoL after RC repair.^37^ Conversely, less negative pain thoughts after RC repair surgery was associated with lower pain, greater satisfaction, and improved functional outcomes, as well as a higher likelihood of reaching clinically meaningful recovery benchmarks such as Patient Acceptable Symptom State and Minimal Clinically Important Difference.^37^ Sabo and colleagues reported that although pain catastrophizing did not significantly change over one year after RC repair, it still contributed to shoulder dysfunction before and after RC repair.^62^ Our results are supported by the findings of a study (n = 33) that reported increased pain level (VAS) in patients with high levels of catastrophizing (PCS) during the first 5 days after RC repair surgery.^67^ Collectively, these findings highlight the need for future research to clarify the role of pain perception including negative pain beliefs and pain catastrophizing in RC repair recovery and to better understand its association with functional outcomes and PA levels.

Like pain-related beliefs, kinesiophobia is infrequently assessed in those before and after RC repair.^17^^,^^73^ In studies that did assess kinesiophobia, we found that post-operative kinesiophobia consistently decreased over time as early as 6 weeks and continuing up to one year following RC repair.^17^^,^^73^ Movement-related fear is clinically relevant as it may contribute to activity avoidance, muscle weakness, limited range of motion, poor functional outcomes, and ultimately reduced PA.^67^^,^^79^ Experiencing kinesiophobia before and after RC repair surgery may increase shoulder dysfunction and pain.^73^ A study showed that RC pathology patients with higher levels of kinesiophobia demonstrated greater shoulder dysfunction and more severe pain compared to those with lower levels of kinesiophobia.^73^ Similar results were reported by Deniz and colleagues,^17^ in which they found that patients with RC pathology experienced kinesiophobia, depression, reduced functional capacity, balance asymmetry, and decreased mobility before repair surgery. Following shoulder surgeries, greater baseline kinesiophobia and pain catastrophizing were found to predict greater pain 3 months after surgery.^69^ These findings highlight the potential importance of early recognition and addressing kinesiophobia before and in the early recovery period following RC repair surgery to support optimal recovery trajectories.

### Limitations pertaining to the current scoping review

Several limitations were identified related to the findings from this scoping review. Firstly, although we conducted a comprehensive literature search among several databases, there remains the possibility that relevant or unpublished studies were missed. Secondly, due to the heterogeneity of the study design, psychological well-being measures reported (eg, HADS, PROMIS, BDI, Tampa Scale of Kinesiophobia), and outcome measures reported (eg, ASES, WORC, SF-12), direct comparisons across studies were not always feasible. However, this variability also highlights the need for more standardized approaches and longitudinal research to better understand the role of psychological well-being and PA in individuals with RC pathology. Thirdly, the use of self-reported patient-reported outcomes (PROs) as the primary outcome measure used across the included studies introduces some potential for recall and social desirability biases,^5^^,^^36^ and the consequent lack of objective shoulder-related data. While PROs are valuable for assessing subjective patient evaluations on pain, function, and psychological factors,^17^ they may not fully capture objective changes in shoulder muscle strength and ROM, which are critical for understanding shoulder functional performance following RC repair. Future studies should consider incorporating objective measures such as muscle strength testing, ROM, and device-measured PA to comprehensively evaluate and measure the magnitude of recovery before and after RC repair surgery.

In addition, the studies included in this scoping review relied on varied follow-up periods (eg, 6 months, 12 months, and 2 years), making it challenging to generalize the findings of this review across studies. Furthermore, studies included were both prospective and retrospective observational study designs, leading to variation in the methodological quality and potential risk of bias between studies.^14^ Additionally, as the included studies in this review were conducted in different countries including Italy, Korea, Canada, Turkey, China, Switzerland, and the United States, cross-cultural differences and variations in surgical and rehabilitation protocols across countries may influence the generalizability of our findings. Because we focused on psychological response to injury and recovery, this may be significantly influenced by sociocultural and health system factors. Lastly, the fact that we could not locate any studies measuring PA before and after RC repair represents an important gap and limitation of this review.

### Clinical implications and future research directions from the current scoping review

The findings of this scoping review indicated a need to integrate psychological well-being assessments and interventions into the care provided to patients undergoing RC repair. Incorporating psychological screening and interventions before RC repair surgery could improve patient outcomes either before or both before and after surgery, as suggested by published evidence from other orthopedic injuries/procedures.^2^^,^^23^^,^^44^ The consistent prevalence of poor psychological well-being including depression, anxiety, and kinesiophobia before RC repair surgery and their association with post-operative measures^17^^,^^27^^,^^31^^,^^41^^,^^73^ warrant them to be considered in pre-operative planning similar to other modifiable pre-operative factors such as body mass index and diabetes control.^11^^,^^60^ Specifically, patients undergoing RC repair surgery may benefit from implementing multidisciplinary techniques (eg, patient education, mindfulness-based techniques, or cognitive behavioral strategies) during pre-operative rehabilitation to potentially improve patient engagement, reduce emotional distress, and promote adherence to rehabilitation protocols.^25^ Lastly, cross-cultural studies are needed to determine the role of cultural context, health systems, and social support in psychological well-being and recovery outcomes in patients undergoing RC repair.

Even though RC pathology is an upper extremity condition that still allows free mobility of the lower extremities, there may still be a clinically relevant impact on PA levels in this population. To our knowledge, only one study has examined patient-reported PA in patients following RC repair.^12^ Whereas this study was not included in the present review because it did not evaluate PA both before and after RC repair, it evaluated PA using the International Physical Activity Questionnaire and found that patients with higher levels of PA 6 weeks after surgery exhibited lower levels of pain, greater shoulder flexion, abduction, and external rotation ROM and better functional recovery measured by ASES compared to those with lower levels of PA^12^. The fact that we could not locate any study that included device- or questionnaire-measured PA assessment before and after RC repair surgery reveals a significant gap in the literature. This highlights the need for future longitudinal studies and randomized controlled trials to explore PA measures before RC repair surgery and PA outcomes after surgery. Moreover, movement-related fear such as kinesiophobia, negative pain beliefs, and pain catastrophizing may be linked to lower levels of PA in patients undergoing RC repair. Understanding these relationships may help to guide more holistic and effective future rehabilitation strategies for patients undergoing RC repairs.

## Conclusion

This scoping review highlights an uneven distribution of research attention across psychological constructs, PA, and outcome measures, highlighting the need for more comprehensive, consistent evaluation approaches. Future research is suggested to explore the interplay between PA levels and psychological factors and their influence on RC repair recovery. Additionally, consideration of potential mediators and confounding factors will be critical in guiding more holistic and effective rehabilitation strategies.
